# Supplementary material for: Thymines opposite to bulky aristolactam-DNA adducts in duplex DNA are not targeted by human thymine-DNA glycosylase
Source: PeerJ. 2025 Jul 4;13:e19577. doi: 10.7717/peerj.19577 (PMC12232928; doi:10.7717/peerj.19577)
Supplement: Supplemental Information 1 [file peerj-13-19577-s001.docx]

**Supplemental Information Table S1**. Statistical analysis of data in Figure 3A,C. The action of TDG^FL^ on dumbbell DNA duplexes containing dA-ALI. Data are presented as mean ± standard deviation (SD) for each cleavage product observed in the gel.

| **Figure** | **Lane N°** | **Reaction** | **Cleavage product (length)** | **Cleavage** ± **SD (%)** |  |
| --- | --- | --- | --- | --- | --- |
| **Fig. 3A** | 2 | 14-03 dmbDNA G•T* + TDG 1h | 25 mer | 10.60±1.10 |  |
|  | 3 | 14-03 dmbDNA Hx•T* + TDG 1h | 25 mer | 8.33±1.86 |  |
|  | 4 | 14-03 dmbDNA G•T* + TDG 1h, 0.1 M NaCl | 25 mer | 7.40±1.95 |  |
|  | 5 | 14-03 dmbDNA Hx•T* + TDG 1h, 0.1 M NaCl | 25 mer | 6.27±1.33 |  |
|  | 9 | 14-03 dmbDNA dA-ALI•T* + TDG 18h | 25 mer | 5.70±4.86 |  |
|  | 10 | 14-03 dmbDNA dA-ALI•T* + TDG 1h, 0.1 M NaCl | 25 mer | 0.15±0.15 |  |
|  | 11 | 14-03 dmbDNA dA-ALI•T* + TDG 6h, 0.1 M NaCl | 25 mer | 0.32±0.19 |  |
|  | 12 | 14-03 dmbDNA dA-ALI•T* + TDG 18h, 0.1 M NaCl | 25 mer | 1.33±0.47 |  |
|  | 7 | 14-03 dmbDNA dA-ALI•T* + TDG 1h | 29 mer | 2.58±0.68 |  |
|  | 8 | 14-03 dmbDNA dA-ALI•T* + TDG 6h | 29 mer | 14.77±2.06 |  |
|  | 9 | 14-03 dmbDNA dA-ALI•T* + TDG 18h | 29 mer | 40.93±17.15 |  |
|  | 10 | 14-03 dmbDNA dA-ALI•T* + TDG 1h, 0.1 M NaCl | 29 mer | 1.37±0.50 |  |
|  | 11 | 14-03 dmbDNA dA-ALI•T* + TDG 6h, 0.1 M NaCl | 29 mer | 3.27±0.71 |  |
|  | 12 | 14-03 dmbDNA dA-ALI•T* + TDG 18h, 0.1 M NaCl | 29 mer | 13.07±3.50 |  |
|  | 7 | 14-03 dmbDNA dA-ALI•T* + TDG 1h | 35 mer | 0.51±0.25 |  |
|  | 8 | 14-03 dmbDNA dA-ALI•T* + TDG 6h | 35 mer | 3.37±0.87 |  |
|  | 9 | 14-03 dmbDNA dA-ALI•T* + TDG 18h | 35 mer | 20.9±21.2 |  |
|  | 10 | 14-03 dmbDNA dA-ALI•T* + TDG 1h, 0.1 M NaCl | 35 mer | 0.31±0.10 |  |
|  | 11 | 14-03 dmbDNA dA-ALI•T* + TDG 6h, 0.1 M NaCl | 35 mer | 0.72±0.17 |  |
|  | 12 | 14-03 dmbDNA dA-ALI•T* + TDG 18h, 0.1 M NaCl | 35 mer | 2.23±0.68 |  |
|  | 7 | 14-03 dmbDNA dA-ALI•T* + TDG 1h | 17 mer | 0.39±0.12 |  |
|  | 8 | 14-03 dmbDNA dA-ALI•T* + TDG 6h | 17 mer | 2.62±1.33 |  |
|  | 9 | 14-03 dmbDNA dA-ALI•T* + TDG 18h | 17 mer | 6.57±4.07 |  |
|  | 10 | 14-03 dmbDNA dA-ALI•T* + TDG 1h, 0.1 M NaCl | 17 mer | 0.27±0.13 |  |
|  | 11 | 14-03 dmbDNA dA-ALI•T* + TDG 6h, 0.1 M NaCl | 17 mer | 0.45±0.15 |  |
|  | 12 | 14-03 dmbDNA dA-ALI•T* + TDG 18h, 0.1 M NaCl | 17 mer | 1.43±0.19 |  |
|  | 7 | 14-03 dmbDNA dA-ALI•T* + TDG 1h | 15 mer | 0.65±0.23 |  |
|  | 8 | 14-03 dmbDNA dA-ALI•T* + TDG 6h | 15 mer | 4.67±1.46 |  |
|  | 9 | 14-03 dmbDNA dA-ALI•T* + TDG 18h | 15 mer | 7.6±4.95 |  |
|  | 10 | 14-03 dmbDNA dA-ALI•T* + TDG 1h, 0.1 M NaCl | 15 mer | 0.88±0.49 |  |
|  | 11 | 14-03 dmbDNA dA-ALI•T* + TDG 6h, 0.1 M NaCl | 15 mer | 1.26±0.84 |  |
|  | 12 | 14-03 dmbDNA dA-ALI•T* + TDG 18h, 0.1 M NaCl | 15 mer | 2.58±0.97 |  |
|  | 7 | 14-03 dmbDNA dA-ALI•T* + TDG 1h | 10 mer | 0.17±0.16 |  |
|  | 8 | 14-03 dmbDNA dA-ALI•T* + TDG 6h | 10 mer | 0.75±0.38 |  |
|  | 9 | 14-03 dmbDNA dA-ALI•T* + TDG 18h | 10 mer | 0.81±0.25 |  |
|  | 10 | 14-03 dmbDNA dA-ALI•T* + TDG 1h, 0.1 M NaCl | 10 mer | 0.14±0.13 |  |
|  | 11 | 14-03 dmbDNA dA-ALI•T* + TDG 6h, 0.1 M NaCl | 10 mer | 0.25±0.13 |  |
|  | 12 | 14-03 dmbDNA dA-ALI•T* + TDG 18h, 0.1 M NaCl | 10 mer | 0.45±0.22 |  |
|  |  |  |  |  |  |
| **Figure** | **Lane N°** | **Reaction** | **Cleavage product (length)** | **Cleavage (%)** |  |
| **Fig. 3C** | 2 | 14-04 dmbDNA G•T* + TDG 1h | 25 mer | 8.10±2.61 |  |
|  | 3 | 14-04 dmbDNA Hx•T* + TDG 1h | 25 mer | 3.03±1.46 |  |
|  | 4 | 14-04 dmbDNA G•T* + TDG 1h, 0.1 M NaCl | 25 mer | 6.57±3.11 |  |
|  | 5 | 14-04 dmbDNA Hx•T* + TDG 1h, 0.1 M NaCl | 25 mer | 0.5±0.2 |  |
|  | 7 | 14-04 dmbDNA dA-ALI•T* + TDG 1h | 25 mer | 0.08±0.03 |  |
|  | 8 | 14-04 dmbDNA dA-ALI•T* + TDG 6h | 25 mer | 0.41±0.13 |  |
|  | 9 | 14-04 dmbDNA dA-ALI•T* + TDG 18h | 25 mer | 3.17±1.31 |  |
|  | 10 | 14-04 dmbDNA dA-ALI•T* + TDG 1h, 0.1 M NaCl | 25 mer | 0.07±0.06 |  |
|  | 11 | 14-04 dmbDNA dA-ALI•T* + TDG 6h, 0.1 M NaCl | 25 mer | 0.22±0.10 |  |
|  | 12 | 14-04 dmbDNA dA-ALI•T* + TDG 18h, 0.1 M NaCl | 25 mer | 2.23±1.01 |  |
|  | 7 | 14-04 dmbDNA dA-ALI•T* + TDG 1h | 29 mer | 2.40±0.82 |  |
|  | 8 | 14-04 dmbDNA dA-ALI•T* + TDG 6h | 29 mer | 10.07±2.20 |  |
|  | 9 | 14-04 dmbDNA dA-ALI•T* + TDG 18h | 29 mer | 34.33±11.53 |  |
|  | 10 | 14-04 dmbDNA dA-ALI•T* + TDG 1h, 0.1 M NaCl | 29 mer | 1.80±0.17 |  |
|  | 11 | 14-04 dmbDNA dA-ALI•T* + TDG 6h, 0.1 M NaCl | 29 mer | 6.13±1.22 |  |
|  | 12 | 14-04 dmbDNA dA-ALI•T* + TDG 18h, 0.1 M NaCl | 29 mer | 20.77±2.46 |  |
|  | 7 | 14-04 dmbDNA dA-ALI•T* + TDG 1h | 35 mer | 0.44±0.15 |  |
|  | 8 | 14-04 dmbDNA dA-ALI•T* + TDG 6h | 35 mer | 2.63±1.04 |  |
|  | 9 | 14-04 dmbDNA dA-ALI•T* + TDG 18h | 35 mer | 17.23±6.75 |  |
|  | 10 | 14-04 dmbDNA dA-ALI•T* + TDG 1h, 0.1 M NaCl | 35 mer | 0.34±0.07 |  |
|  | 11 | 14-04 dmbDNA dA-ALI•T* + TDG 6h, 0.1 M NaCl | 35 mer | 1.42±0.60 |  |
|  | 12 | 14-04 dmbDNA dA-ALI•T* + TDG 18h, 0.1 M NaCl | 35 mer | 8.70±4.47 |  |
|  | 7 | 14-04 dmbDNA dA-ALI•T* + TDG 1h | 17 mer | 0.20±0.10 |  |
|  | 8 | 14-04 dmbDNA dA-ALI•T* + TDG 6h | 17 mer | 1.79±1.32 |  |
|  | 9 | 14-04 dmbDNA dA-ALI•T* + TDG 18h | 17 mer | 5.67±0.75 |  |
|  | 10 | 14-04 dmbDNA dA-ALI•T* + TDG 1h, 0.1 M NaCl | 17 mer | 0.12±0.10 |  |
|  | 11 | 14-04 dmbDNA dA-ALI•T* + TDG 6h, 0.1 M NaCl | 17 mer | 0.64±0.29 |  |
|  | 12 | 14-04 dmbDNA dA-ALI•T* + TDG 18h, 0.1 M NaCl | 17 mer | 2.60±0.66 |  |
|  | 7 | 14-04 dmbDNA dA-ALI•T* + TDG 1h | 15 mer | 0.57±0.47 |  |
|  | 8 | 14-04 dmbDNA dA-ALI•T* + TDG 6h | 15 mer | 2.40±0.70 |  |
|  | 9 | 14-04 dmbDNA dA-ALI•T* + TDG 18h | 15 mer | 10.30±2.39 |  |
|  | 10 | 14-04 dmbDNA dA-ALI•T* + TDG 1h, 0.1 M NaCl | 15 mer | 0.29±0.11 |  |
|  | 11 | 14-04 dmbDNA dA-ALI•T* + TDG 6h, 0.1 M NaCl | 15 mer | 1.73±0.50 |  |
|  | 12 | 14-04 dmbDNA dA-ALI•T* + TDG 18h, 0.1 M NaCl | 15 mer | 6.50±1.23 |  |
|  | 7 | 14-04 dmbDNA dA-ALI•T* + TDG 1h | 10 mer | 0.07±0.06 |  |
|  | 8 | 14-04 dmbDNA dA-ALI•T* + TDG 6h | 10 mer | 0.27±0.21 |  |
|  | 9 | 14-04 dmbDNA dA-ALI•T* + TDG 18h | 10 mer | 1.23±1.05 |  |
|  | 10 | 14-04 dmbDNA dA-ALI•T* + TDG 1h, 0.1 M NaCl | 10 mer | 0.07±0.12 |  |
|  | 11 | 14-04 dmbDNA dA-ALI•T* + TDG 6h, 0.1 M NaCl | 10 mer | 0.17±0.29 |  |
|  | 12 | 14-04 dmbDNA dA-ALI•T* + TDG 18h, 0.1 M NaCl | 10 mer | 0.75±0.49 |  |

**Supplemental Information Table S2**. Statistical analysis of data in Figure 4. The action of TDG^FL^ on different strands of dA-ALI•T* dmbDNA. Data are presented as mean ± standard deviation (SD) for each cleavage product observed in the gel.

| **Figure** | **Lane N°** | **Reaction** | **Cleavage product (length)** | **Cleavage (%)** |  |
| --- | --- | --- | --- | --- | --- |
| **Fig. 4A** | 2 | 14-03 dmbDNA G•T* + TDG 1h | 25 mer | 10.6±1.1 |  |
|  | 4 | 14-03 dmbDNA dA-ALI•T* + TDG 1h | 25 mer | 0.3±0.3 |  |
|  | 5 | 14-03 dmbDNA dA-ALI•T* + TDG 18h | 25 mer | 5.70±4.86 |  |
|  | 4 | 14-03 dmbDNA dA-ALI•T* + TDG 1h | 29 mer | 2.58±0.68 |  |
|  | 5 | 14-03 dmbDNA dA-ALI•T* + TDG 18h | 29 mer | 40.93±17.15 |  |
|  | 4 | 14-03 dmbDNA dA-ALI•T* + TDG 1h | 35 mer | 0.51±0.25 |  |
|  | 5 | 14-03 dmbDNA dA-ALI•T* + TDG 18h | 35 mer | 20.9±21.2 |  |
|  | 4 | 14-03 dmbDNA dA-ALI•T* + TDG 1h | 17 mer | 0.39±0.12 |  |
|  | 5 | 14-03 dmbDNA dA-ALI•T* + TDG 18h | 17 mer | 6.57±4.07 |  |
|  | 4 | 14-03 dmbDNA dA-ALI•T* + TDG 1h | 15 mer | 0.65±0.23 |  |
|  | 5 | 14-03 dmbDNA dA-ALI•T* + TDG 18h | 15 mer | 7.6±4.95 |  |
|  | 7 | 14-03 dmbDNA dA-ALI*•T + TDG 1h | 10 mer | 0.23±0.15 |  |
|  | 8 | 14-03 dmbDNA dA-ALI*•T + TDG 18h | 10 mer | 1.70±0.72 |  |
|  |  |  |  |  |  |
| **Figure** | **Lane N°** | **Reaction** | **Cleavage product (length)** | **Cleavage (%)** |  |
| **Fig. 4C** | 10 | 14-04 dmbDNA G•T* + TDG 1h | 25 mer | 8.10±2.61 |  |
|  | 12 | 14-04 dmbDNA dA-ALI•T* + TDG 1h | 25 mer | 0.08±0.03 |  |
|  | 13 | 14-04 dmbDNA dA-ALI•T* + TDG 18h | 25 mer | 3.17±1.31 |  |
|  | 12 | 14-04 dmbDNA dA-ALI•T* + TDG 1h | 29 mer | 2.40±0.82 |  |
|  | 13 | 14-04 dmbDNA dA-ALI•T* + TDG 18h | 29 mer | 34.33±11.53 |  |
|  | 12 | 14-04 dmbDNA dA-ALI•T* + TDG 1h | 35 mer | 0.44±0.15 |  |
|  | 13 | 14-04 dmbDNA dA-ALI•T* + TDG 18h | 35 mer | 17.23±6.75 |  |
|  | 12 | 14-04 dmbDNA dA-ALI•T* + TDG 1h | 17 mer | 0.20±0.10 |  |
|  | 13 | 14-04 dmbDNA dA-ALI•T* + TDG 18h | 17 mer | 5.67±0.75 |  |
|  | 12 | 14-04 dmbDNA dA-ALI•T* + TDG 1h | 15 mer | 0.57±0.47 |  |
|  | 13 | 14-04 dmbDNA dA-ALI•T* + TDG 18h | 15 mer | 10.30±2.39 |  |
|  | 15 | 14-04 dmbDNA dA-ALI*•T + TDG 1h | 16 mer | 1.53±0.40 |  |
|  | 16 | 14-04 dmbDNA dA-ALI*•T + TDG 18h | 16 mer | 20.30±3.08 |  |
|  |  |  |  |  |  |

**Supplemental Information Table S3**. Statistical analysis of data in Figure 5. Removal of bulky dA-ALI adduct in the top 27-mer strand of dA-ALI*•T dmbDNA through combined action of TDG^FL^ and APE1. Data are presented as mean ± standard deviation (SD) for each cleavage product observed in the gel.

| **Figure** | **Lane N°** | **Reaction** | **Cleavage product (length)** | **Cleavage (%)** |  |
| --- | --- | --- | --- | --- | --- |
| **Fig. 5A exo** | 2 | 14-03 dmbDNA G•T17* + TDG 1h | 16 mer | 1.22±0.51 |  |
|  | 4 | 14-04 dmbDNA A•T* + TDG 1h LP | 16 mer | 0.59±0.14 |  |
|  | 5 | 14-04 dmbDNA A•T* + TDG 1h 5 nM APE1 | 16 mer | 0.21±0.06 |  |
|  | 6 | 14-04 dmbDNA A•T* + TDG 1h 20 nM APE1 | 16 mer | 0.09±0.04 |  |
|  | 7 | 14-04 dmbDNA A•T* + TDG 1h 50 nM APE1 | 16 mer | 0.35±0.48 |  |
|  | 8 | 14-04 dmbDNA A•T* + TDG 1h 200 nM APE1 | 16 mer | 0 |  |
|  | 10 | 14-04 dmbDNA dA-ALI*•T + TDG 1h LP | 16 mer | 1.12±0.28 |  |
|  | 11 | 14-04 dmbDNA dA-ALI*•T + TDG 1h 5 nM APE1 | 16 mer | 1.50±0.36 |  |
|  | 12 | 14-04 dmbDNA dA-ALI*•T + TDG 1h 20 nM APE1 | 16 mer | 1.57±0.21 |  |
|  | 13 | 14-04 dmbDNA dA-ALI*•T + TDG 1h 50 nM APE1 | 16 mer | 1.18±0.32 |  |
|  | 14 | 14-04 dmbDNA dA-ALI*•T + TDG 1h 200 nM APE1 | 16 mer | 0 |  |
|  | 16 | 14-04 U17* UNG 1 nM 10 min | 16 mer | 94.8±3.0 |  |

**Supplemental Information Table S4**. Statistical analysis of data in Figure 6. The action of TDG^FL^ on short blunt-end duplex oligonucleotides containing A, dA-ALI or dA-ALII. Data are presented as mean ± standard deviation (SD) for each cleavage product observed in the gel.

| **Figure** | **Lane N°** | **Reaction** | **Cleavage product (length)** | **Cleavage (%)** |  |
| --- | --- | --- | --- | --- | --- |
| **Fig. 6A** | 2 | 10-05 24 mer G•T* + TDG 1h | 10 mer | 31.13±7.16 |  |
|  | 4 | 10-05 24 mer A•T* + TDG 1h | 10 mer | 2.85±0.73 |  |
|  | 5 | 10-05 24 mer A•T* + TDG 18h | 10 mer | 8.90±1.28 |  |
|  | 5 | 10-05 24 mer A•T* + TDG 18h | 14 mer | 0.20±0.20 |  |
|  | 7 | 10-05 24 mer A*•T + TDG 1h | 10 mer | 14.47±6.55 |  |
|  | 8 | 10-05 24 mer A*•T + TDG 18h | 10 mer | 44.50±8.49 |  |
|  | 10 | 10-05 24 mer G•U* + TDG 10 min | 14 mer | ± |  |
|  | 12 | 10-05 24 mer dA-ALII•T* + TDG 1h | 14 mer | 0.16±0.15 |  |
|  | 13 | 10-05 24 mer dA-ALII•T* + TDG 18h | 14 mer | 6.67±1.39 |  |
|  | 13 | 10-05 24 mer dA-ALII•T* + TDG 18h | 10 mer | 1.0±0.4 |  |
|  | 15 | 10-05 24 mer dA-ALII*•T + TDG 1h | 10 mer | 0.12±0.11 |  |
|  | 16 | 10-05 24 mer dA-ALII*•T + TDG 18h | 10 mer | 5.10±1.49 |  |
|  |  |  |  |  |  |
| **Fig. 6C** | 2 | 14-07 24 mer G•T* + TDG 1h | 10 mer | 9.37±2.59 |  |
|  | 4 | 14-07 24 mer A•T* + TDG 1h | 10 mer | 2.27±0.83 |  |
|  | 5 | 14-07 24 mer A•T* + TDG 18h | 10 mer | 8.73±1.07 |  |
|  | 5 | 14-07 24 mer A•T* + TDG 18h | 14 mer | 0.15±0.15 |  |
|  | 7 | 14-07 24 mer dA-ALI•T* + TDG 1h | 14 mer | 0.65±0.38 |  |
|  | 8 | 14-07 24 mer dA-ALI•T* + TDG 18h | 14 mer | 10.07±1.65 |  |
|  | 8 | 14-07 24 mer dA-ALI•T* + TDG 18h | 10 mer | 0.29±0.26 |  |
|  | 10 | 14-07 24 mer dA-ALI*•T + TDG 1h | 10 mer | 0.97±0.31 |  |
|  | 11 | 14-07 24 mer dA-ALI*•T + TDG 18h | 10 mer | 7.27±1.82 |  |

**Supplemental Information Table S5**. Statistical analysis of data in Figure 7. The action of TDG^FL^ on short blunt-end duplex oligonucleotides containing A, dA-ALI or dG-ALII. Data are presented as mean ± standard deviation (SD) for each cleavage product observed in the gel.

| **Figure** | **Lane N°** | **Reaction** | **Cleavage product (length)** | **Cleavage (%)** |  |
| --- | --- | --- | --- | --- | --- |
| **Fig. 7A** | 2 | 14-06 19 mer G13•T* + TDG 1h | 6 mer | 24.77±6.47 |  |
|  | 4 | 14-06 19 mer A•T* + TDG 1h | 10 mer | 0.15±0.13 |  |
|  | 5 | 14-06 19 mer A•T* + TDG 18h | 10 mer | 3.50±1.54 |  |
|  | 7 | 14-06 19 mer A*•T + TDG 1h | 7 mer | 0.25±0.22 |  |
|  | 8 | 14-06 19 mer A*•T + TDG 1h | 7 mer | 2.20±0.56 |  |
|  | 10 | 14-06 19 mer dA-ALI•T* + TDG 1h | 10 mer | 0.11±0.10 |  |
|  | 11 | 14-06 19 mer dA-ALI•T* + TDG 18h | 10 mer | 7.43±2.26 |  |
|  | 13 | 14-06 19 mer dA-ALI*•T + TDG 1h | 7 mer | 0.04±0.05 |  |
|  | 14 | 14-06 19 mer dA-ALI*•T + TDG 18h | 7 mer | 0.54±0.54 |  |
|  | 16 | 14-06 19 mer G9•T* + TDG 1h | 10 mer | 96.47±1.75 |  |
|  |  |  |  |  |  |
| **Fig. 7C** | 2 | 10-13 24 mer G•T* + TDG 1h | 14 mer | 91.83±4.80 |  |
|  | 4 | 10-13 24 mer A•T* + TDG 1h | 10 mer | 6.73±1.30 |  |
|  | 5 | 10-13 24 mer A•T* + TDG 18h | 10 mer | 18.63±3.53 |  |
|  | 7 | 10-13 24 mer A*•T + TDG 1h | 10 mer | 4.05±1.64 |  |
|  | 8 | 10-13 24 mer A*•T + TDG 18h | 10 mer | 15.21±2.41 |  |
|  | 10 | 10-13 24 mer dG-ALII•T* + TDG 1h | 10 mer | 3.60±1.05 |  |
|  | 11 | 10-13 24 mer dG-ALII•T* + TDG 18h | 10 mer | 55.23±10.19 |  |
|  | 13 | 10-13 24 mer dG-ALII*•T + TDG 1h | 12 mer | 0.87±0.90 |  |
|  | 14 | 10-13 24 mer dG-ALII*•T + TDG 18h | 12 mer | 2.43±0.60 |  |
|  | 16 | 10-13 24 mer G9•T* + TDG 1h | 10 mer | 2.53±0.67 |  |
