# Supplementary figures and images for: Thymines opposite to bulky aristolactam-DNA adducts in duplex DNA are not targeted by human thymine-DNA glycosylase
Source: PeerJ. 2025 Jul 4;13:e19577. doi: 10.7717/peerj.19577 (PMC12232928; doi:10.7717/peerj.19577)

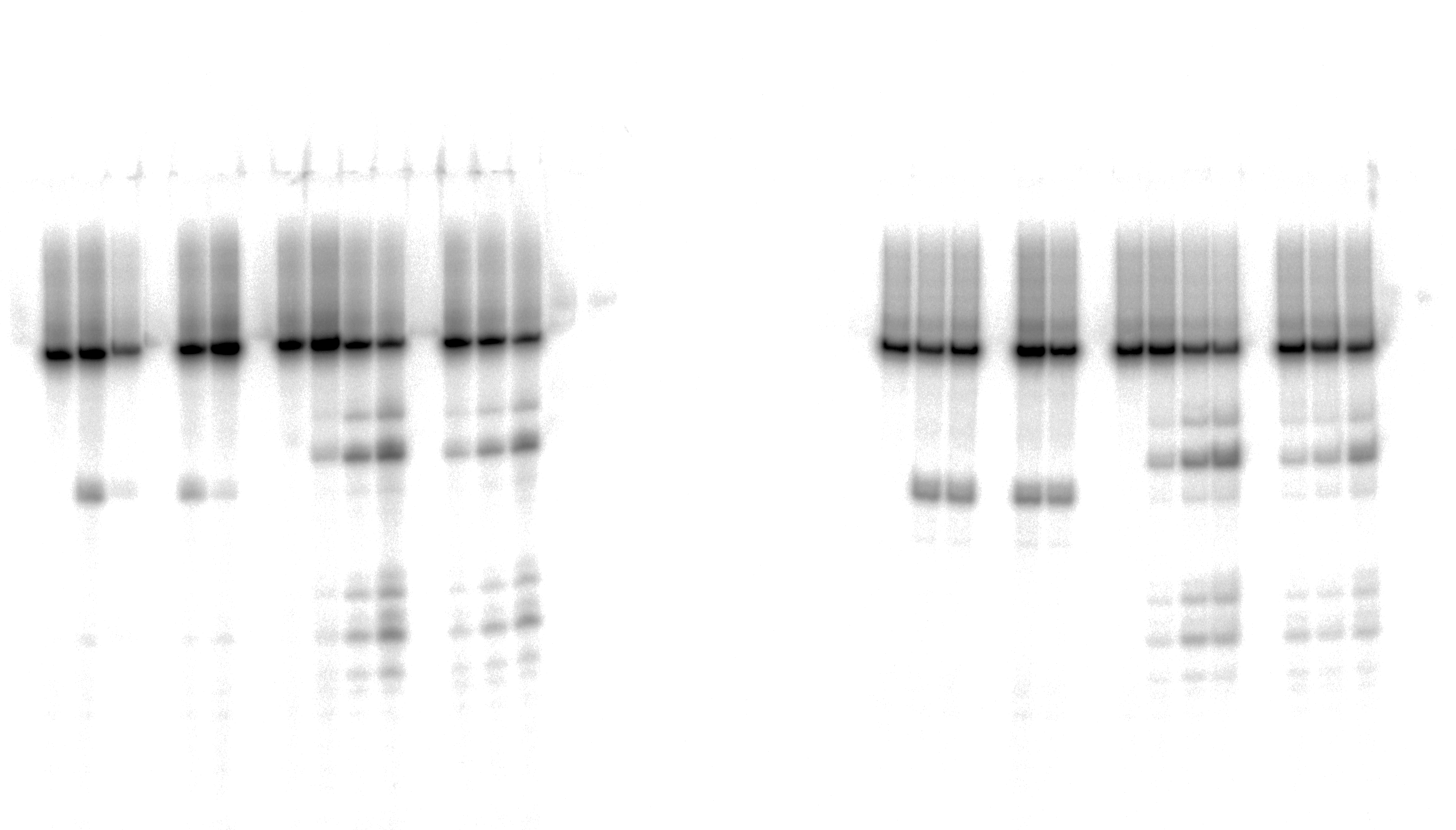

Supplement: Supplemental Information 2 — The action of TDGFL on dumbbell DNA duplexes containing dA-ALI. [file peerj-13-19577-s002.png]

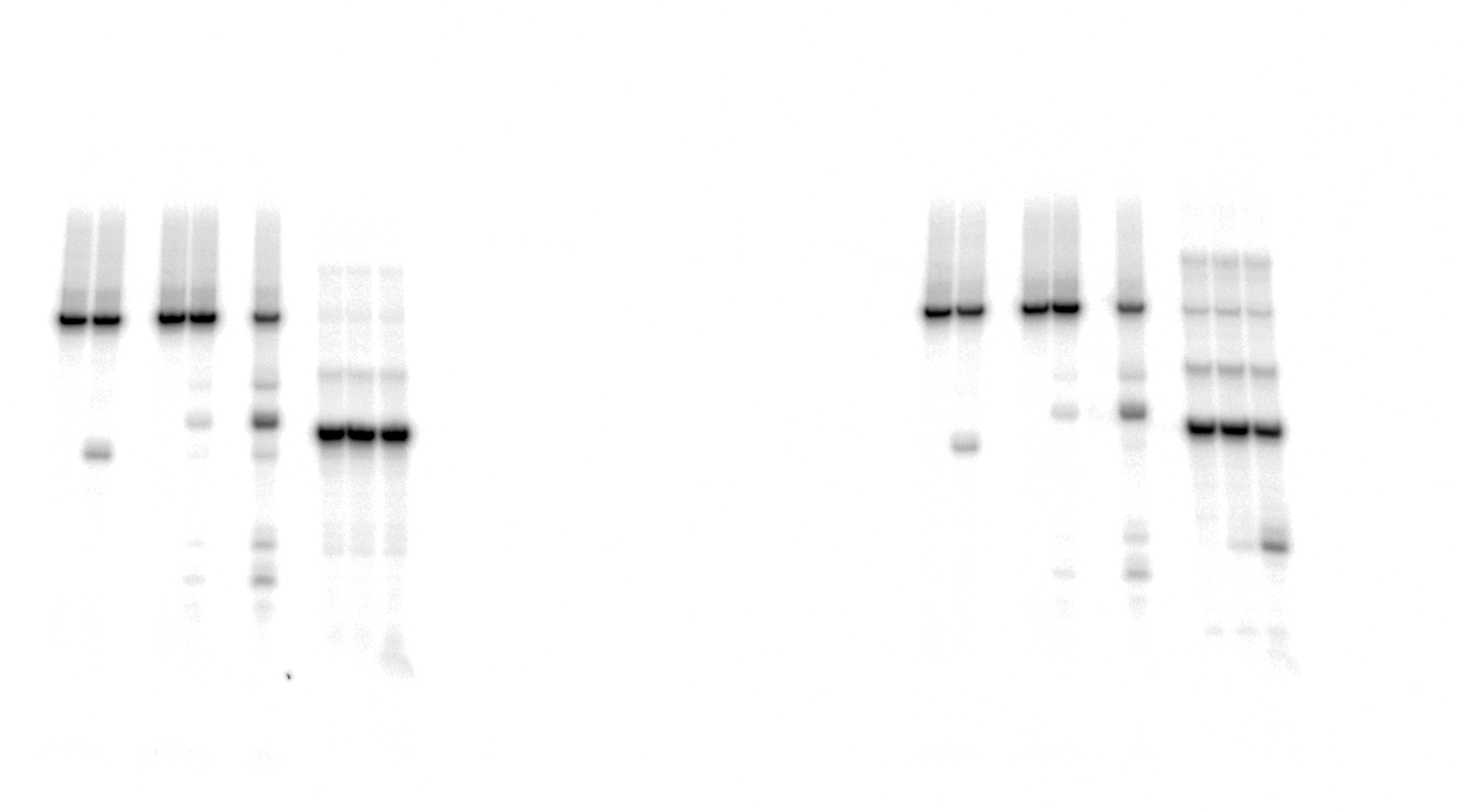

Supplement: Supplemental Information 3 — The action of TDGFL on different strands of dA-ALI•T* dmbDNA. [file peerj-13-19577-s003.png]

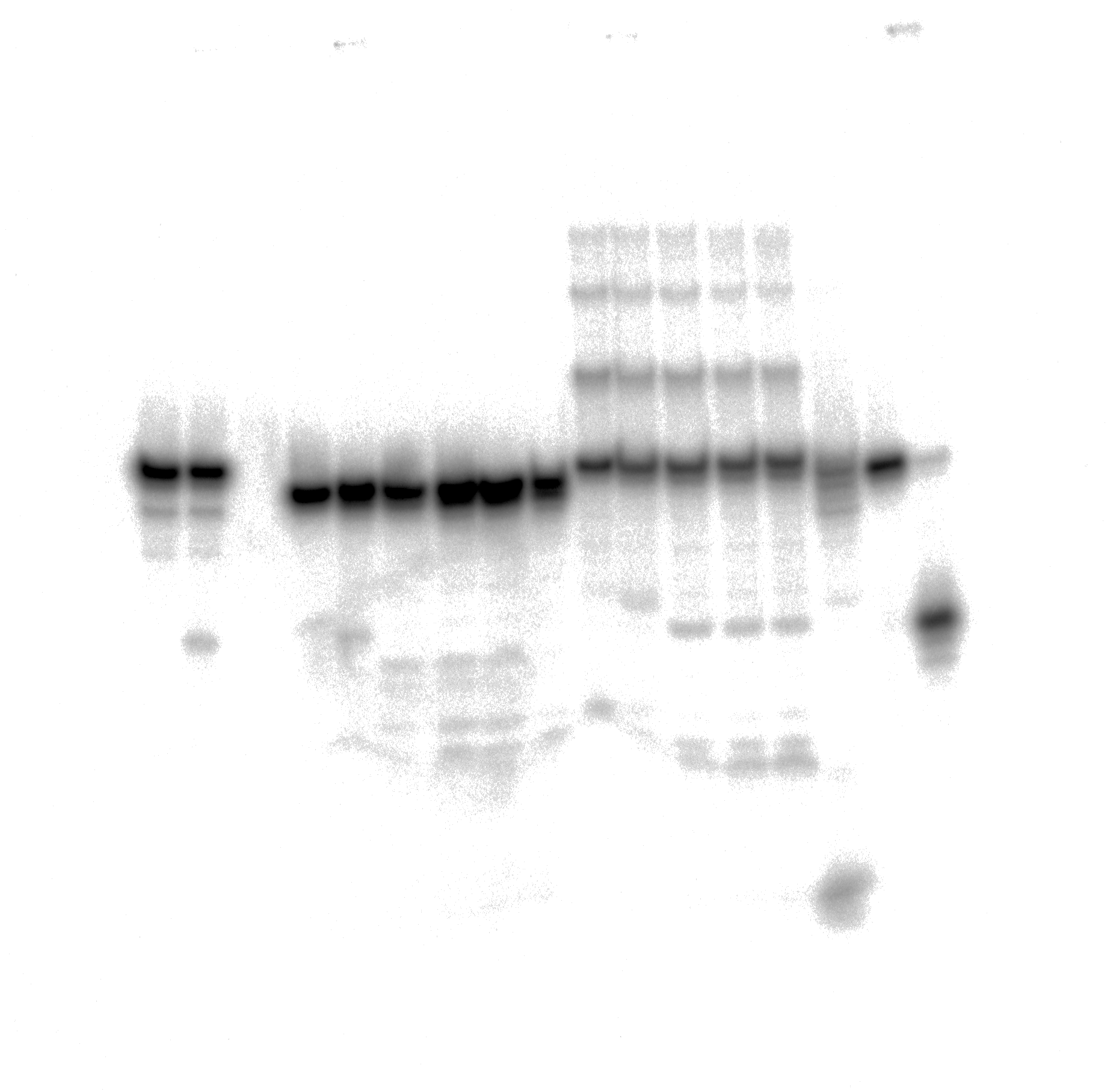

Supplement: Supplemental Information 4 — Removal of bulky dA-ALI adduct in the top 27-mer strand of dA-ALI*•T dmbDNA through combined action of TDG FL and APE1. [file peerj-13-19577-s004.png]

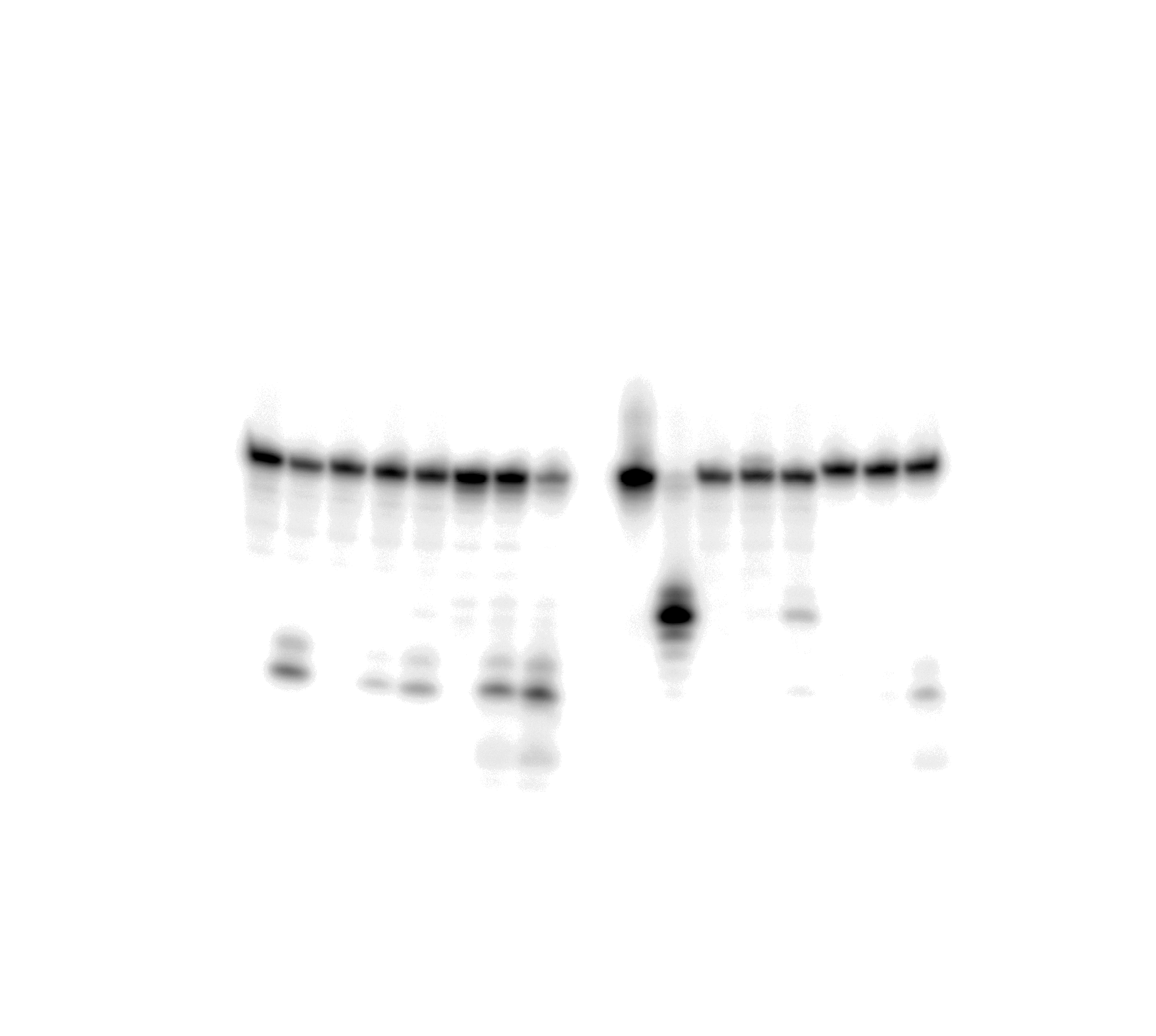

Supplement: Supplemental Information 5 — The action of TDG FL on short blunt-end duplex oligonucleotides containing A, dA-ALI or dA-ALII. [file peerj-13-19577-s005.png]

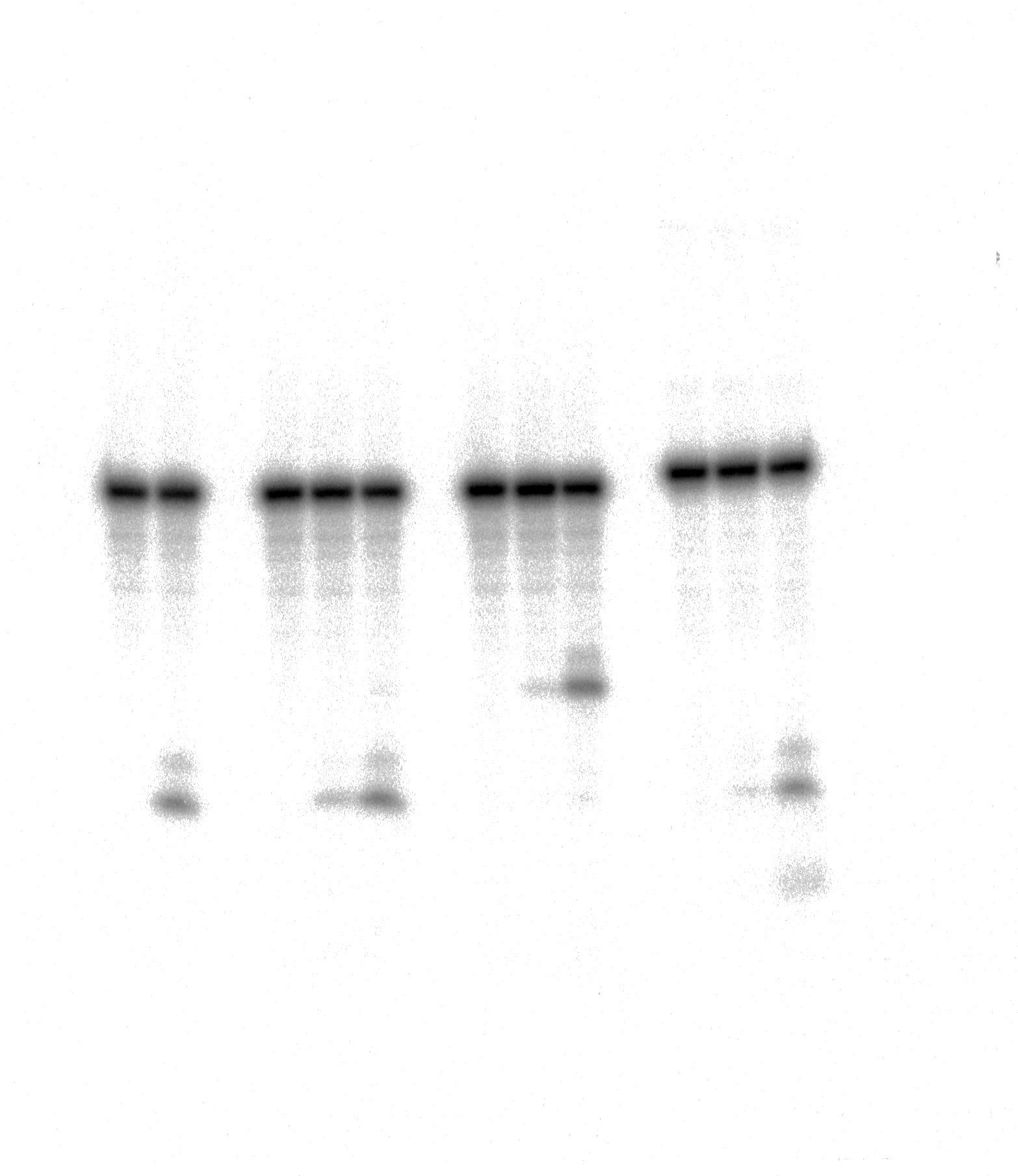

Supplement: Supplemental Information 6 — The action of TDG FL on short blunt-end duplex oligonucleotides containing A, dA-ALI or dA-ALII. [file peerj-13-19577-s006.png]
